# The development and testing of the TTU food cue reactivity image bank

**DOI:** 10.1038/s41366-025-01856-9

**Published:** 2025-07-26

**Authors:** William R. Quarles, Martin Binks

**Affiliations:** 1 Department of Nutritional Sciences, Texas Tech University, Lubbock, TX USA; 2 Department of Nutrition and Food Studies, George Mason University, Fairfax, VA USA

**Keywords:** Nutrition, Obesity

## Abstract

**Background/Objectives:**

Measuring food cue reactivity (FCR) is essential to understanding human ingestion in behavioral and neuroimaging studies. An image bank that can delineate the truly food-specific FCR response from spurious noise driven primarily by incidental characteristics (e.g., visual properties) of images is a useful addition to the literature. This study sought to develop and test the performance of the TTU Food Cue Reactivity Image Bank that matched 252 image pairs on visual characteristics (i.e., shape, color, visual complexity, and size), and to establish the appeal ratings of the images for use in future applications.

**Subjects/Methods:**

The TTU Food Cue Reactivity Image Bank was initially created, and subsequently evaluated by independent raters. Then, 151 individuals participated in a Qualtrics survey to determine the similarity of image pairs on relevant dimensions (i.e., shape, color, visual complexity, and size) and establish appeal ratings for food and non-food images.

**Results:**

Inter-rater agreement was tested using intraclass correlation coefficients (ICC), which revealed very high agreement among raters for all similarity measures (shape ICC = 0.98; color ICC = 0.97; visual complexity ICC = 0.96; size ICC = 0.96; appeal ICC = 0.93).

**Conclusions:**

The high agreement among raters on the visual characteristics and appeal ratings of the images increases confidence that food-cue-reactivity observed is based on intended “image type” distinctions (i.e., food vs. object) and not incidental visual features.

## Introduction

Food cue reactivity (FCR) approaches examine an individual’s response to particular aspects of the food environment [1]. Common applications include functional magnetic resonance imaging (fMRI) [2–6] and behavioral [7–9] studies. The visual FCR paradigm involves exposing individuals to images of foods and non-foods to contrast their responses [1–5, 10–13], and produces a more robust response than gustatory or olfactory cues [14]. Visual FCR is often used in neuroimaging studies because of ease of application in the context of the MRI compared to other methods (e.g., olfactory, gustatory).

This study aims to develop and test a novel FCR image bank (i.e., TTU Food Cue Reactivity Image Bank) that maintains salient image characteristics that are relevant to the human experience of food (e.g., visually recognizable, not scrambled) while eliminating incidental stimuli (i.e., shape, color, visual complexity, and size) that often add noise to FCR studies. For these visual dimensions, we focus on subjective impressions of similarity, because pairwise similarity judgments are often more successful at capturing processes related to higher-level categorization than physical stimulus dimensions, which may not be independently meaningful or evaluable by participants [15–17]. Furthermore, we will provide normative ratings for all relevant aspects of the dataset that are based on subjective impressions of appeal, shape, color, visual complexity, and size. By providing detailed and specific information related to incentive salience and controlling for incidental image characteristics (i.e., shape, color, size, visual complexity) our image bank will allow for improved precision in identifying the behavioral and neurophysiological activations and reduce incidental activations.

Our image bank uniquely combines essential characteristics of existing banks into one comprehensive resource, presenting relevant parameters for users to tailor image subsets to their unique purposes when designing studies. Here we will summarize the contributions of prior work in conceptualizing and developing our image bank.

Several studies have considered visual characteristics during visual FCR presentations, including image descriptions and selection criteria. However, these studies typically do not make the images available nor do they present detailed analyses related to visual characteristics [10, 11, 18–20]. Others have shared image banks online or published analyses related to visual characteristics of food images [21–23], but do not include non-food images, limiting their broader utilization across various types of studies. When end users require matched non-food images, they must add them, which can be time-consuming, especially if matching based on secondary characteristics is needed. A few studies have included the selection criteria for matching food and non-food images, but did not validate the selection by publishing visual characteristic analyses, or only used computer vision tools [24, 25]. Several groups have considered visual perception and image characteristics, and published validation results [12, 13, 26–32], which are summarized in Table 1.

**Table 1:** Summary of validated food image banks.

Image bank	Reference(s)	Image types	Validation dimensions	Image considerations and matching
**KUMC Image Bank** (Kansas Univ. Medical Center)	Szabo-Reed et al. (2015, 2020)	Professional stock images of unpaired foods and non-foods (animals, blurred objects)	Valence, arousal, and appetitive data using human participants	Images matched on brightness, resolution, and size
**Food-Pics**	Blechert et al. (2014, 2019)	Unpaired food and non-food images obtained from a commercially available database	Familiarity, recognizability, complexity, valence, arousal, palatability, and desire to eat	Images uniformly matched on resolution and background. Computer vision properties (i.e., RGB values, brightness, contrast, complexity, luminance, spectral power) were analyzed
**Full4Health Image Collection**	Charbonnier et al. (2016)	4 groups of food images (i.e., low calorie sweet, high calorie sweet, low calorie savory, high calorie savory) and non-food images	Recognizability, liking, perceived healthiness, and perceived number calories using human survey data of adults and children	Images generated using a standardized food photographing protocol
**FoodCast Research Image Database (FRIDa)**	Foroni et al. (2013)	8 categories: natural food, transformed food, rotten food, artificial food objects, natural nonfood, artificial objects, animals, and scenes	Valence, arousal, familiarity, and typicality collected from human survey data	Computed vision properties (i.e., visual ambiguity, size, brightness, and high spatial frequency power) are provided, but not matched
**Open Library of Affective Foods (OLAF)**	Miccoli et al. (2014)	Food images obtained from restaurants and homemade meals	Valence, arousal, dominance, and food craving collected from survey data on adolescents	
**Food-Cal**	Shankland et al. (2019)	Paired food and non-food images obtained from a commercially available database	Attractiveness, arousal, palatability	Foods and non-foods were selectively matched on global shape, range of colors, luminance, and resolution. Computer vision properties (i.e., luminance, contrast, structure) were used to create the structural similarity index

A limitation of the current FCR literature is the lack of consistent methods for selecting and presenting food and non-food images. Some studies abandon the use of non-food images in favor of using most and least appealing food images as rated by the individual participant as a comparator [19, 20]. While this method accounts for individual preferences in food, it cannot account for activation related to the visual characteristics of the image independent of the food being rated. One method for choosing non-food images is to use common strategies for creating “control” images in vision science such as phase scrambling [33], or “diffeomorphic” transformations [34] on the selected food images. These methods have the limitation of not creating ideal controls for FCR because meaningful objects can evoke a wide range of semantic and affective processes that would not be evoked by meaningless objects with similar visual properties [35]. Indeed, food not only has meaning but also can vary, from image to image, on incentive salience, a key measure FCR approaches are intended to isolate.

Some efforts have been made to balance semantic meaning by comparing food images to images of tools [11] or animals [10, 12, 13, 18], but these categories still likely cover only a limited range as compared with affective processing of food [36]. It is possible to control for this by selecting a variety of non-foods versus any single non-food category. Thus, greater variation in non-foods is critical for triangulating the true effect of food [6] in semantic space. Moreover, comparing food images to a single semantic category has the inherent weakness of not being able to find the food-specific response that FCR studies are intended to isolate. In fMRI FCR, the use of the subtraction method where activation from food is subtracted from non-food finds brain regions that activate when distinguishing between foods and the semantic category, rather than finding the effect of food itself. Indeed, it is just as possible that differences in activation are due to the inverse effect being present in the semantic category. Therefore, if the study finds differential activation in one region of the brain during the food > object comparison, it is just as likely that region is simply not used in processing of that object as it is in processing food. Thus, several previous approaches cannot answer whether the observed FCR responses are driven primarily by the visual aspects of an image, differences in incentive salience, differences in meaningfulness, or a true food-specific response.

Overall, the existing literature provides several options that are suited for specific types of approaches. However, as a whole there are some gaps that our image bank addresses in a single image bank that can also be used to develop subsets of images based on specific study parameters. For example, our image bank provides rigorous control of several factors that may result in spurious behavioral and/or neurophysiological activations. It also provides realistic (unscrambled) food and object images which are needed in many FCR studies (as opposed to phase-scrambled). Also, our images provide information related to hedonic properties (low to high appeal) of images and offer a wide range (low to high appeal) for both food and non-food images. These characteristics of our image bank are important in designing FCR protocols to address specific study needs in studies examining realistic visual cues. Additionally, if a study requires phase-scrambled images our images can provide base stimuli for this purpose with the benefits outlined above incorporated. The image bank presented here is not designed to necessarily replace the well-constructed image banks currently available, however it provides a relatively large set of well-controlled, versatile, and validated images with accompanying data that will assist many. It may be particularly useful given the rapidly expanding interest in FCR by providing a readily available option for those first starting out.

## Methods

### Ethical considerations

The study was approved by the Texas Tech University Institutional Review Board (IRB) as exempt based on anonymous data collection and minimal risk to subjects. All methods were performed in accordance with the relevant guidelines and regulations. Participants were provided informed consent on the survey landing page and given the option to proceed or discontinue. All methods and analyses were pre-specified prior to image bank creation, and all data-driven analyses are discussed herein.

### Image bank creation

The study team consisting of the PI, 1 study staff member and 2 graduate research assistants developed an intuitive search strategy to seek a wide range of cooked and uncooked foods that would be representative of a “typical” western diet. The search started broadly with major meal categories entered as keywords (e.g., Breakfast, Lunch, Dinner, and Snack Foods). Images were selected based on the judgment of the study team member according to the general criteria of: high image quality, able to isolate the food from the photo’s background, relevance to the study of FCR involving a typical western diet. As images were accumulated, the study team collectively reviewed the images to ensure representation of a wide range of food groups from a variety of caloric densities, tastes, portion sizes, and subjective hedonic values. Thereafter, focused searches were conducted to find specific foods identified as missing and necessary. Non-food images to be paired with food images were then obtained using the reverse image search function in Google Images. Keywords were added if the reverse image search function could not find a suitable non-food image. Images were confirmed as not having specific, explicit copyright protection against use in research under the fair use doctrine [37] before downloading.

**Fig. 1:**
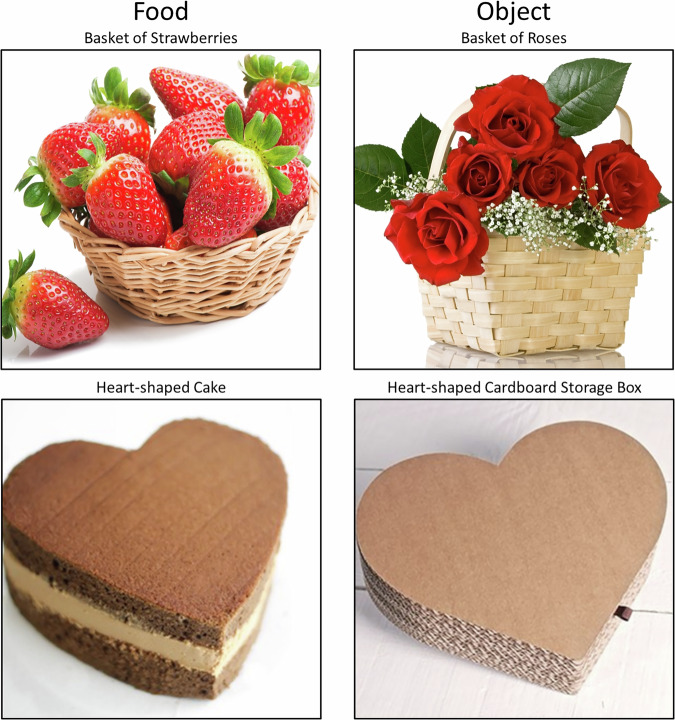
Example pairs of food and non-food (basket of roses and basket of strawberries, heart-shaped cake and heart-shaped cardboard storage box).

Image pairs were chosen based on similarity of shape, color, visual complexity, and size (i.e., size within visual field of the image), and were lightly edited (e.g., cropping) if necessary. The result was pairs of food and non-food items that appeared similar (e.g., a basket of roses and a basket of strawberries, a heart-shaped cake and a heart-shaped cardboard storage box; Fig. 1). All image pairs are freely available within a data repository at Open Science Framework found at https://osf.io/8s25t/.

All images were checked for quality and similarity by at least 2 members of the research team. If images were deemed unsatisfactory during the quality check, replacement images were found. After compilation of images was complete, the image bank was evaluated by an independent rater for similarity in shape, color, visual complexity, and size, and to ensure that food and non-food images could be readily identified as such. All images were then converted to 96.00 ppi on both the x- and y-axes, scaled to 150 × 150 pixels, and saved as JPEG files via GNU Image Manipulation Program (GIMP) [38]. If necessary, images were further edited using GIMP to maximize similarity measures.

### Participants

**Fig. 2:**
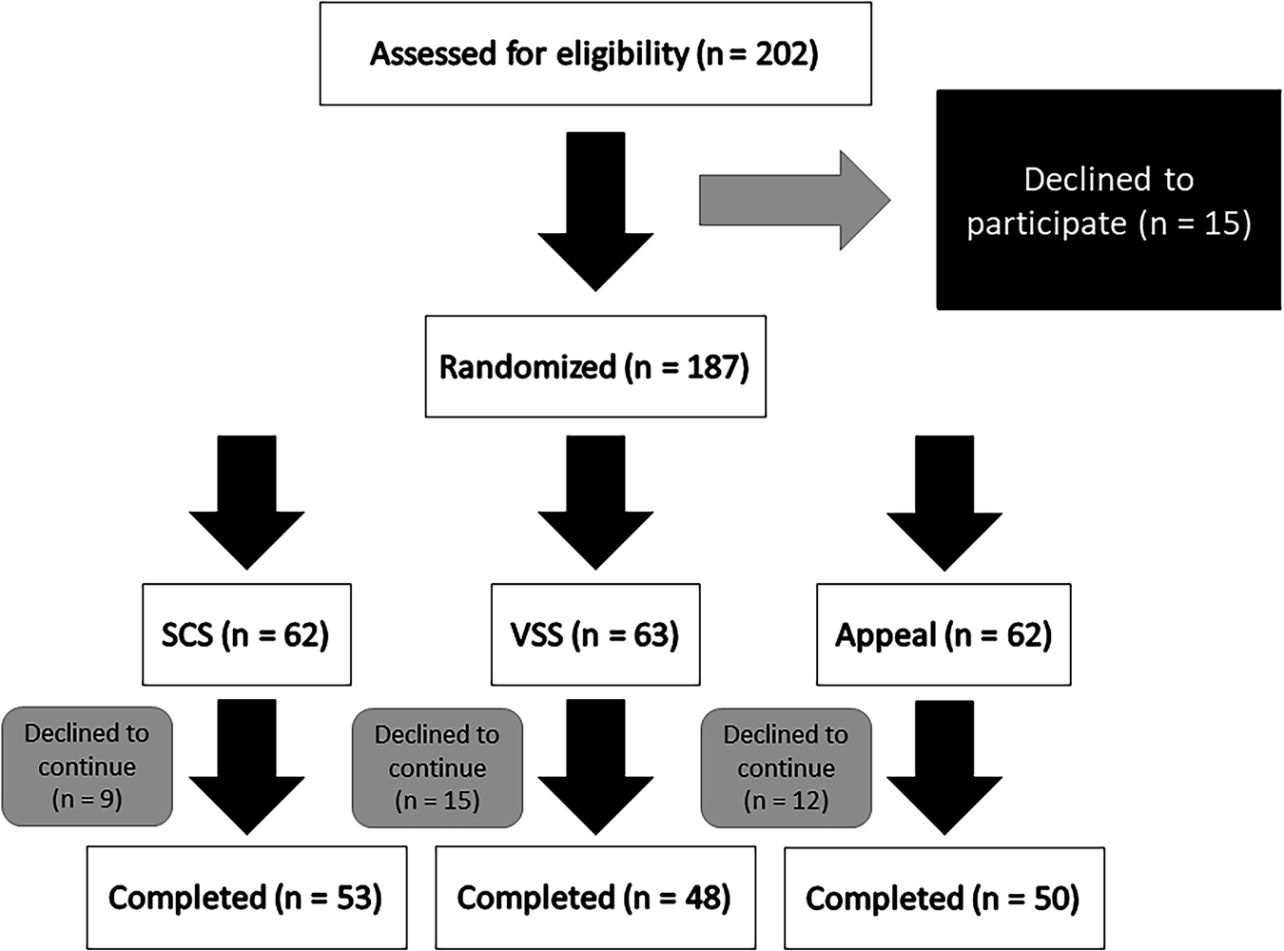
Eligibility and randomization of study participants and completion of data collection (SCS Shape and Color Similarity, VSS Visual Complexity and Size Similarity).

Two hundred and two adults (age 18 years or older) were assessed for eligibility, and 151 completed the Qualtrics [39] survey designed to collect data to determine the similarity of image pairs on relevant dimensions (i.e., shape, color, visual complexity, and size) and establish appeal ratings for food and non-food images. Recruitment of subjects was completed by posting a Human Intelligence Task (HIT) on Amazon Mechanical Turk (MTurk). Users of MTurk are representative of the United States general population, with the exceptions that they are younger, more educated, and more likely to be of White or Asian ethnicity when compared to the U.S. population [40]. For completing the study, subjects were compensated $2 after verifying HIT completion on MTurk. Details of the sample used in this study can be found in the CONSORT-Like diagram (Fig. 2).

### Image bank performance testing

Subjects participated via a mobile or non-mobile device using a supported browser (i.e., Safari, Chrome, Edge, Internet Explorer, and Firefox). Potential subjects interested in participating responded to the HIT and were redirected to the Qualtrics survey. After being provided with study information, potential subjects responded to demographic information, and were automatically screened for study age requirements. After meeting the age requirement and answering demographic questions, subjects were randomized into one of three groups: Shape and Color Similarity (SCS), Visual Complexity and Size Similarity (VSS), and Appeal. The SCS group rated each of the 252 image pairs on a 0–100 Likert scale in agreement with the prompt: “These 2 images have a similar overall shape.” They then rated each of the 252 image pairs on a 0–100 Likert scale in agreement with the prompt: “These 2 images have similar colors.” The VSS group rated each of the 252 image pairs on a 0–100 Likert scale in agreement with the prompt: “These 2 images have a similar amount of details.” They then rated each of the 252 image pairs on a 0–100 Likert scale in agreement with the prompt: “These 2 images are a similar size.” The Appeal group rated each of the 504 individual images on a 7-point Likert scale answering the question “How appealing is this to you?” Images / image pairs displayed during rating were randomized using the Randomized function in Qualtrics. Inter-subject agreement was tested using intra-class correlations for appeal ratings and similarity values (i.e., shape, color, visual complexity, and size). Statistical analyses were completed using the irr package [41] in R (version 3.3.2) [42].

### Image characteristics

To further test the similarity of image pairs, we computed relevant image properties. We computed red, green, and blue (RGB) color channels using the EBImage package [43] in R (version 3.3.2), and calculated luminance using the product of these three color channels. To test for the difference between paired and non-paired images, all values for all images were put into a matrix. Euclidean distances (ED) were calculated for all possible images. Statistical analyses were then conducted using Welch’s *t* tests to compare the ED of all matched images compared to all possible combinations of non-matched images.

## Results

One hundred and fifty-one male and female participants (*n* = 70, *n* = 81, respectively) with mean age of 35 years (SD = 10.2) and mean BMI 26.9 ((SD = 6.87) kg/m^2^) completed the survey. A high degree of reliability was found between all similarity measures and appeal ratings. Analysis of average intraclass correlations are reported in Table 2. Specific similarity and appeal ratings for each image pair can be found in S1 Table in the supplementary information.

**Table 2:** Reliability of similarity scale and appeal ratings.

	Intraclass correlation	95% CI	F	*P*
**Shape Similarity**	0.98	[0.98, 0.98]	F(251, 13052) = 51.8	<2 × 10^−16^
**Color Similarity**	0.97	[0.97, 0.98]	F(251, 13052) = 37.4	<2 × 10^−16^
**Complexity Similarity**	0.96	[0.95, 0.97]	F(251, 11797) = 24.1	<2 × 10^−16^
**Size Similarity**	0.96	[0.96, 0.97]	F(251, 11797) = 26.4	<2 × 10^−16^
**Appeal Ratings**	0.93	[0.92, 0.94]	F(503, 24144) = 14.3	<2 × 10^−16^

Paired images were more alike using computational image properties than non-paired images. Analysis of ED of image properties are reported in Table 3. Specific RGB and luminance values for each image pair can be found in S2 Table in the supplementary information.

**Table 3:** Euclidean distances of computational image properties.

Image Property	Average ED of paired images	Average ED of non-paired images	*P*
**All Distances**	0.26	0.53	<2 × 10^−16^
**Red**	0.11	0.19	<2 × 10^−16^
**Green**	0.11	0.23	<2 × 10^−16^
**Blue**	0.13	0.28	<2 × 10^−16^
**Luminance**	0.13	0.28	<2 × 10^−16^

## Discussion

This study sought to develop and test the performance of the TTU Food Cue Reactivity Image Bank. Findings indicated a high degree of agreement for all similarity constructs and appeal ratings. This confirms that efforts to appropriately match images on incidental characteristics (i.e., shape, color, visual complexity, and size) and incentive values (i.e., appeal ratings) were successful. In short, a significant confound found throughout the literature whereby the influence of the incidental properties of the stimuli is indistinguishable from the intended measurement target (i.e., food cue reactivity) [44–46] has been accounted for successfully in this image bank.

### Strengths and limitations

The study and resultant image bank with published normative data can contribute to both behavioral and neuroimaging FCR literature in several ways. First, by matching image pairs on secondary perceptual visual characteristics (i.e., shape, color, size, visual complexity) this image bank substantially improves upon other published image banks and addresses a salient issue seen in the literature. For example, the food-pics database [26, 27], using an approach without complete sets of matched food and non-food images, fails to account for potential variability and influences of subjective visual properties and incongruent incentive salience of food and non-food images. These issues are addressed a priori based on the methodology used in developing and testing the TTU FCR image bank, removing some of the ‘noise’ created by image properties that interfere with the primary goal of identifying food-non-food-based differences.

Second, this is the first publicly available image bank to account for food appeal. Instead of relying on beliefs about properties of foods as they relate to hedonic value (e.g., caloric density), the current study utilizes measured values (i.e., appeal ratings) to directly gather information related to perceived hedonic value of the images via appeal ratings measured during the actual stimulus presentation. Additionally, our image bank attends to the hedonic qualities of both food and non-food images, the latter being largely ignored in the current literature that typically assumes neutrality in incentive salience of the non-food images. Given the diversity of factors that influence appeal (for food and objects) it is important to quantify this in an image bank. Clearly certain objects may invoke variable hedonic responses (e.g., a sports car vs. a comb) that may need to be accounted for in some food—object contrasts. By quantifying this for both food and objects in our image bank, and providing that data to researchers, we eliminate the typical practice of inferring hedonic valence based on inherent societal understanding that may introduce bias. Ultimately, these data will allow the end user to design study parameters around a known level of “appeal” and introduce specificity as needed (in both neuroimaging and behavioral studies).

Third, to our knowledge this is the first rigorously tested, public domain FCR image bank to measure perceived image similarity. The availability of this performance-tested, validated image bank and the methods used to develop it will encourage standardization of image attributes in the FCR literature moving forward. The availability will additionally remove burden from those entering the field by providing an already tested image bank and availability of detailed methodology will allow others to apply these techniques in expanding, developing and testing their own. The result being improved consistency and interpretive coherence across FCR studies and the potential facilitation of comparisons used in systematic reviews and meta-analyses.

With the publication of the actual images and normative values of salient domains, future users of this image bank can be confident that the behavioral and neurophysiological outcomes of interest they are measuring are not influenced by incidental factors related to the nature of the images. Furthermore, this will allow for customization and further performance testing, meeting the specific needs of each study. Beyond using the full image bank, image pairs can be selectively used to develop image subsets based on specific similarity and appeal ratings. For instance, if a study aimed to focus on the response to ‘berries’ during a food cue reactivity study, all images containing berries could be selected from this image bank, knowing that image pairs were already matched on color, shape, visual complexity, and size. Similarly, if a study was interested in only a subset of high- or low- appeal foods, image pairs could be selectively chosen that contain a high food/object appeal rating. Given the large number of images in this image bank (252 image pairs) there would likely be a sufficient number of image pairs to meet the needs of such subsets. If more images were needed, the methods delineated here form the basis for evaluating an unlimited expansion of the number of images. Ultimately, establishing the similarity of images in our image bank has the advantage of enabling the end-user to utilize the images and associated data in any way that is beneficial for their scientific study.

One example of utilization of our image bank is by sorting or blocking on any dimension where the stimuli have been measured. Because matching based on high-level visual information lends itself more to event-related designs that is how we have primarily employed the image bank. However, if comparing individual image pairs is not of interest, other designs such as the historically common block design work as well. A blocked design could retain the benefits of our visual pairs by organizing stimulus trains so foods have the same order as their paired non-foods across a block. Another example of image bank utilization is by categorization. If an investigator determined caloric content of our images and wanted to compare images of high-calorie foods, low-calorie foods, and non-food objects, they would simply split the image bank based on high-calorie foods and their non-food pairs, and low-calorie foods with their non-food pairs. To find the effect of caloric content, the investigator would use the following formula: (high-calorie food - their non-food matches) - (low-calorie food - their non-food matches). This is useful because visual variance is still removed due to image matching. This same formula could be used for any dimension an investigator would want to sort on. The only limitation is that it would not allow for the simple, direct comparison of: high-calorie food image – low-calorie food image. However, this limitation is minimal because visual similarity has already been adjusted for.

For end users interested in generating custom subsets of images based on dimensional matching data (Supplementary Table 1), we recommend two primary strategies to filter for high-quality matches. The first strategy is to set a minimum threshold for each similarity dimension (e.g. selecting image pairs that scored at least 50/100 on all or certain dimensions) or using rank-based exclusion (e.g. removing the 10 lowest similarity image pairs for each dimension). The second strategy is to calculate a composite distance metric (e.g. Cook’s distance, Euclidean distance) to create a single image similarity score and remove the least similar pairs overall. Research teams may also combine these methods to ensure their subset meets both general and dimension-specific criteria for image similarity (e.g. first applying a minimum threshold to the distance metric and then selectively removing image pairs that exhibit low similarity on any individual dimension).

One limitation of our image bank is variability in the level of matching on relevant properties. In attempting to create a realistic set of food and object images that are applicable to the real world, there are challenges in matching. While imperfect, our image bank makes significant strides to improve upon this to the extent possible without creating overly artificial images. Furthermore, by publishing detailed data related to these parameters, researchers will be able to make informed decisions when selecting subsets of the image bank based on their needs. We anticipate that future users of the image bank will select images according to the specific parameters of interest to their study and their selective tolerance for variability in specific known (published) parameters and/or to control for relevant parameters (e.g., appeal value) in statistical modeling.

Also, developing a model that allows for contrasting similarity ratings of image pairs as compared to similarity ratings of non-matched image pairs is beneficial. By comparing pairwise similarities of all images, a multidimensional representational space could be created, and could in turn be used to compare matched image pairs to one another, similar to previously published work in perception [47]. This would add to the current study by giving a clearer idea of how well the image pairs are visually matched, as the current study only obtained ratings for image pairs that were intended to be matched based on the initial selection. However, obtaining pairwise ratings between 504 objects (from 252 pairs) would be prohibitive in any single set of subjects as the number of unique pairs is large (126,756).

An additional limitation is that our image bank does not represent all relevant demographics. The study was normalized on users of MTurk, and the image bank may need to be normalized on the demographic if our image bank is used to study another population group.

A final area of improvement would be to add an overall similarity rating to the dimensions measured. Currently, we cannot know how important each of the visual characteristics are for the totality of the rated similarity of an image pair. Thus, a systematic analysis of the degree to which each individual similarity rating (i.e., shape, color, visual complexity, and size) contributes to the overall similarity rating may provide meaningful insights into the specific elements in the subjective FCR experience of the individual.

## Conclusion

In conclusion, the TTU Food Cue Reactivity Image Bank is an important addition to the current FCR literature as it addresses several known limitations of the currently available image banks in the FCR literature. Furthermore, making this methodology and the resultant image bank freely available at Open Science Framework (https://osf.io/8s25t/) will contribute in a meaningful way to future studies.

## Supplementary information

supplementary material legends

Supplementary Table 1

Supplementary Table 2

## Data Availability

Data described in the manuscript, code book, and analytic code, as well as images, will be made available upon request.
