# Supplementary material for: The development and testing of the TTU food cue reactivity image bank
Source: Int J Obes (Lond). 2025 Jul 26;49(10):2019–25. doi: 10.1038/s41366-025-01856-9 (PMC12532690; doi:10.1038/s41366-025-01856-9)
Supplement: Supplementary file 1 — supplementary material legends [file 41366_2025_1856_MOESM1_ESM.docx]

Supplementary Table 1. Specific similarity and appeal ratings for image pairs

Supplementary Table 2. Specific Red, Green, and Blue (RGB) and Luminance values for image pairs
